# Iterated Residual Graph Convolutional Neural Network for Personalized Three-Dimensional Reconstruction of Left Myocardium from Cardiac MR Images

**DOI:** 10.3390/s23177430

**Published:** 2023-08-25

**Authors:** Xuchu Wang, Yue Yuan, Minghua Liu, Yanmin Niu

**Affiliations:** 1 Key Laboratory of Optoelectronic Technology and Systems of Ministry of Education, College of Optoelectronic Engineering, Chongqing University, Chongqing 400044, China; 2 College of Computer and Information Science, Chongqing Normal University, Chongqing 400050, China

**Keywords:** 3D reconstruction of the left myocardium, residual graph convolutional neural network, triangular mesh, point cloud

## Abstract

Three-dimensional reconstruction of the left myocardium is of great significance for the diagnosis and treatment of cardiac diseases. This paper proposes a personalized 3D reconstruction algorithm for the left myocardium using cardiac MR images by incorporating a residual graph convolutional neural network. The accuracy of the mesh, reconstructed using the model-based algorithm, is largely affected by the similarity between the target object and the average model. The initial triangular mesh is obtained directly from the segmentation result of the left myocardium. The mesh is then deformed using an iterated residual graph convolutional neural network. A vertex feature learning module is also built to assist the mesh deformation by adopting an encoder–decoder neural network to represent the skeleton of the left myocardium at different receptive fields. In this way, the shape and local relationships of the left myocardium are used to guide the mesh deformation. Qualitative and quantitative comparative experiments were conducted on cardiac MR images, and the results verified the rationale and competitiveness of the proposed method compared to related state-of-the-art approaches.

## 1. Introduction

As one of the most important organs of the human body, the heart plays an essential role in the entire blood circulatory system. Among the various tissues of the heart, the left myocardium is the most important because it pumps blood sent to the entire body via the left ventricle. In addition, many cardiac diseases, such as cardiac hypertrophy, directly manifest as structural abnormalities of the left myocardium. The study of the left myocardium, therefore, holds important pathological significance [1]. Three-dimensional (3D) reconstructions of the left myocardium can be used to accurately calculate the left ventricular volume and other related functional parameters, offering doctors a real three-dimensional sense to assist in the diagnosis and treatment of heart diseases, especially when used with medical virtual reality (VR). However, 3D reconstructions of the left myocardium present challenges due to individual differences and because the heart is constantly moving, which is a personalized variable.

### 1.1. Related Works

For 3D modeling of the left ventricle, traditional methods can be roughly divided into two categories. The first is the use of professional modeling software, such as Maya, AutoCAD, etc., to perform 3D modeling of the heart. However, these methods require modeling personnel to have professional knowledge of computer graphics. In addition, the shape parameters of some irregular soft tissues are too small to be captured, and the accuracy of the model largely depends on the professional experience of the operator. The second method is to perform 3D modeling on acquired two-dimensional cardiac slices (such as CT images, MRI images, etc.) using a specifically designed reconstruction algorithm. These algorithm-based modeling methods can be further subdivided into model-based and non-model-based approaches. Model-based algorithms use a prior model to deform and approximate the shape of the target object. The model is generally a statistical model generated from many acquired data sets. The typical representative methods of this division include the active shape model (ASM) [2] and the active appearance model (AAM) [3] and their improvements. In the ASM model, the average model of the training data is obtained through Procrustes analysis. The weight parameters are then changed so that the average model approximates the surface of the target object. However, the disadvantage of the ASM is that it does not consider the variations in gray levels across different images, whereas the AAM is an improvement of the ASM, which not only considers the shape information but also takes the gray-level information of the image into account [4]. In practice, the accuracy of the model-based algorithms largely depends on how similar the shape of the target object is to the average model, so a model reconstructed using these methods usually lacks general applicability.

On the contrary, non-model-based methods do not rely on any prior shape information; the reconstructed 3D model is completely dependent on the medical image data. Representative algorithms include contour reconstruction, finite element, and marching cubes (MC). For example, Gonzales et al. [5] used a finite element algorithm to reconstruct the atrium of the heart in 3D space, where 142 finite elements were used for the reconstruction of the left atrium, and 90 finite elements were used for the reconstruction of the right atrium. Nugroho et al. [6] reconstructed a 3D heart model using rendering technology and the MC algorithm based on 2D cardiac CT images. The above algorithms consist of hand-designed reconstruction rules. For some methods that cannot be expressed using mathematical formulas, the generalization ability of the model is inevitably restricted. In addition, the accuracy of reconstructed results also depends on the image data.

In recent years, the powerful representation and learning ability of deep learning techniques have attracted researchers to apply them in the field of 3D reconstruction. Eigen et al. [7] applied deep learning to the field of 3D reconstruction for the first time. They directly estimated the depth of a single image with the help of a neural network. The proposed network consists of two parts: one is used to estimate the overall structure of the scene, whereas the other uses local information to refine the prediction results of the first part. It then applies the scale-invariant error function as the loss function to train the entire network.

According to the geometric representation of the reconstructed model, deep-learning-based 3D reconstruction can be roughly divided into three categories: voxel-based, point-cloud-based, and triangular-mesh-based. Voxel-based reconstruction algorithms directly extend the correlation operations from 2D images to 3D voxels. For example, Choy et al. [8] proposed a 3D-R2N2 model for 3D reconstruction from single or multiple 2D images. The model consists of three parts: an encoder, a 3D LSTM, and a decoder. The final reconstruction results are represented by voxels. However, in order to improve the accuracy of the reconstruction, it is necessary to improve the resolution of the reconstruction results, and, accordingly, the amount of computation and storage also greatly increases. Compared with voxels, point clouds have the advantages of relatively small storage and easier manipulation. Based on these advantages, Fan et al. [9] pioneered the application of point clouds in 3D reconstruction using deep learning. They proposed a point cloud generation network, training it using the Chamfer distance loss and earth mover’s distance loss. However, since the points in a point cloud are discrete, they cannot fully express the surface information of the object.

As another representation of the reconstruction results, triangular meshes have the advantages of being lightweight and preserving topology, in contrast to voxels. Furthermore, compared with point clouds, the points in triangular meshes are connected by edges, so the relationships among points are more compact, thus they can better represent the surface information of the object. For example, Wang et al. [10] used an ellipsoid triangle mesh as the initial triangular mesh. The constructed network is divided into two parts: one part is used to extract image features, whereas the other is used to deform the triangle mesh. According to the intrinsic matrix of the camera, the vertices of the triangular mesh are projected onto the feature map of the image. The image features corresponding to the vertices of the triangular mesh are then obtained to assist in the deformation of the initial triangular mesh. In view of the advantages of triangular meshes, this paper also uses triangular meshes to reconstruct the left ventricle from MRI slices.

Compared with traditional methods, a model constructed by deep learning usually needs a large data set and complex training processes. Some methods tried to combine two above categories. For example, Bhalodia et al. [11] proposed a general framework by combining statistical shape modeling and deep learning, which can directly model raw images in a 3D space. The network generates an M-dimensional vector from the original image for subsequent reconstruction. This M-dimensional vector obeys a Gaussian distribution, and correspondingly, there is an average value in the distribution. However, as mentioned above, the accuracy of the reconstructed model by such model-based algorithms is affected by the similarity of the target object to the average model [12]. Some other interesting methods have also been proposed to generate meshes directly from 2D images [13].

### 1.2. Motivation and Contribution

Based on the above analysis, a non-model-based left myocardium 3D reconstruction algorithm is proposed in this paper by designing an iterated residual graph convolution neural networks. The initial triangular mesh is obtained directly from a coarse result generated based on 3D segmentation images. The obtained mesh is then deformed using a residual graph convolutional neural network. Meanwhile, a point feature extraction module is proposed to extract the corresponding point cloud of initial triangular mesh to obtain the characteristic point cloud. Feature fusion with the points in the mesh is then performed to assist the mesh deformation. In an iterative manner, the triangular mesh is adjusted for a better representation of the left myocardium.

Specifically, the main contributions of the proposed method are as follows.

(1) A deep-learning-based approach is applied to the left myocardium 3D reconstruction from MRI slices. The specific residual graph convolution neural network is designed towards mesh deformation with iteration. In 3D reconstruction tasks, the mainstream is based on explicit approaches such as marching cubes or implicit approaches such as Poisson-based reconstruction. However, these methods do not incorporate the point’s feature to guide the mesh deformation. The proposed model focuses on the deep feature learning approach to drive the mesh deformation, making the reconstructed result robust to a variety of shapes and sizes of the left myocardium across different subjects.

(2) A two-stage mesh generation is designed to build a personalized surface of the left myocardium. In the coarse deformation step, the Cubify algorithm [14] is adopted to generate the initial triangular mesh. Compared with the standard marching cubes algorithm, the Cubify approach is simple and fast because it directly converts a voxel into a triangular mesh with fixed triangular faces, edges and vertices. However, the initial triangular mesh obtained by this algorithm is full of sharp edges and corners, and even voxe-wise steps, which do not precisely reflect the real structure of the surface. Therefore, in the fine deformation step, the initial triangular mesh is deformed using a residual graph convolutional neural network to correct the position of each vertex in an iterated manner.

(3) A vertex feature learning module is built to assist the mesh deformation of the left myocardium. The characteristics of the initial points are learned by an encoder–decoder neural network to represent the skeleton of the left myocardium at different receptive fields. In this way, the shape and local relationship of the left myocardium expressed by the deep feature is helpful for subsequent mesh deformation. After that, the corresponding deep feature associated with the vertices on the initial triangular mesh is fused with those on the input triangular mesh of each module in the residual graph convolutional neural network to guide the mesh deformation.

The remainder of this paper is organized as follows. Section 2 describes the details of the proposed framework, which includes initial mesh generation, vertex feature learning and iterated mesh deformation modules using a residual graph convolutional neural network. Section 3 presents the experimental results including data set, experimental platform, evaluation metric, parameter settings, experimental results and discussion with comparison to related state-of-the-art methods. Finally, Section 4 concludes this work and offers its future research potential.

## 2. Proposed Method

### 2.1. Overview

**Figure 1:**
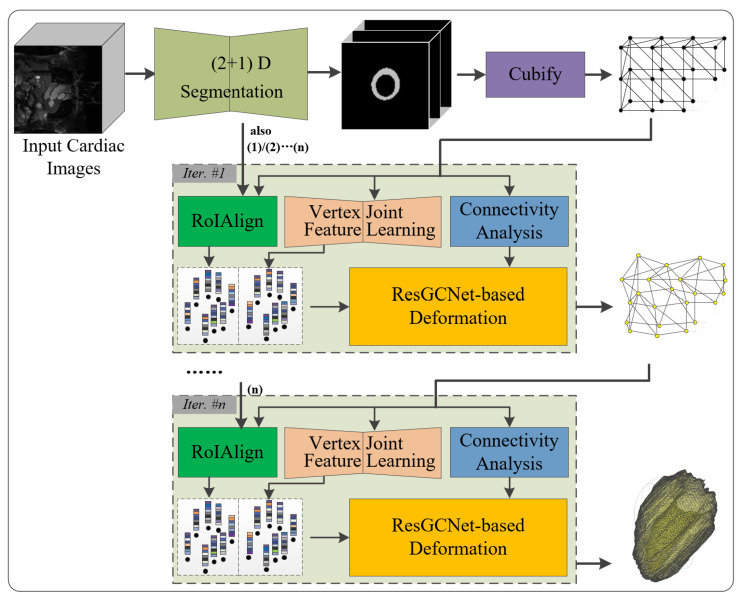
Overall framework of proposed 3D reconstruction of the left myocardium from MR images.

The overall framework of the proposed 3D reconstruction of the left myocardium is shown in Figure 1. The method is based on residual graph convolutional network with deep feature learning and cascaded refinement. Specifically, the framework consists of three modules as follows.

(1) Initial mesh generation module: It builds an initial mesh of the left myocardium using an UNet++ 2.5D segmentation and the Cubify algorithm on the stacked 2D segmented images.

(2) Vertex joint feature learning module: The encoder–decoder neural network is designed to extract the joint deep features of the point cloud and multiple CNN feature maps corresponding to the initial triangular mesh to obtain a skeleton of vertices.

(3) Mesh deformation module: An iterated residual graph convolutional neural network is designed to gradually deform the initial triangular mesh. Meanwhile, the hybrid deep features obtained in the vertex joint feature learning module are added to enrich the features of vertices in each intermediate triangular mesh for obtaining a better reconstruction. As a result, the mesh is rapidly refined with each iteration.

In the following subsections, the details of these modules are presented.

### 2.2. Initial Mesh Generation Module

The geometric structure of the left myocardium varies widely across different people. Even in the same subject at diverse time points of cardiac motion, the shape of the left myocardium muscle changes remarkably. To handle this challenge, the structure of triangular meshes instead of voxels from the 2.5D segmentation results of the left myocardium was suitable to represent the surface.

Typical segmentation methods include traditional multi-atlas label fusion or deep-learning-based methods. Since our work focuses on the 3D reconstruction of the left myocardium, we directly applied the well-known UNet++ based algorithm [15] to produce the segmentation result. Considering the computational burden of 3D convolution in the network, we adopted a (2 + 1)D convolution strategy that focuses on the image to be segmented but with the addition of the two nearest slices in the stack. In this way, the obtained reconstruction results were specific to the individual’s anatomy. In order to obtain the initial triangular mesh faster and easier, the Cubify algorithm [14] was adopted to convert each voxel into a triangular mesh with 12 triangular faces, 18 edges and 8 vertices. Post-processing on the mesh to simplify the triangular facets was then performed according to the following rules: Common faces of adjacent triangle meshes were deleted, common vertices were merged into one vertex, and common edges were merged into a single edge. After that, deformation of the obtained initial triangular mesh was undertaken to obtain the refined reconstruction result.

### 2.3. Vertex Feature Learning Module

The initial triangular mesh obtained by the Cubify algorithm can interpret the shape characteristics of the object; however, it is difficult to accurately recreate the surface of the left myocardium because the mesh contains many abrupt edges and corners. To solve this problem, we propose to optimize the initial surface using a mesh deformation technique based on a graph convolutional neural network. To deform the shape of triangular mesh in the correct direction, it is essential to learn the approximate three-dimensional structural features of the object from its corresponding point cloud of the initial triangular mesh.

Compared to data with regular structure such as images, point cloud data have some undesirable characteristics: irregularity, disorder, and permutation invariance [16,17,18]. These features all bring difficulties to the processing (e.g., feature extraction, object classification) of point cloud data. To handle this, Huang et al. [19] converted point cloud data into voxels, then fed the resulting voxel data into a 3D fully convolutional neural network for voxel-level segmentation. After that, it was assumed that the points located in the same voxel have the same category, thus obtaining the characteristics of each point in the point cloud. However, such methods are limited by the resolution of voxel data and easily introduce artifacts during the voxelization process. High-resolution voxel data require more storage and more computation cost, while lower resolution lead to loss of accuracy.

On the other hand, Lawin et al. [20] used a virtual camera to project point cloud data onto a two-dimensional plane from different angles so as to obtain multiple image data in different angular directions. After that, an image segmentation network was used to process the obtained image data. Finally, the image segmentation results of different angles and directions were fused to obtain the features of each point in the 3D point cloud data. Unfortunately, such methods cannot fully exploit the underlying geometric and structural features of 3D point cloud data, resulting in information loss.

**Figure 2:**
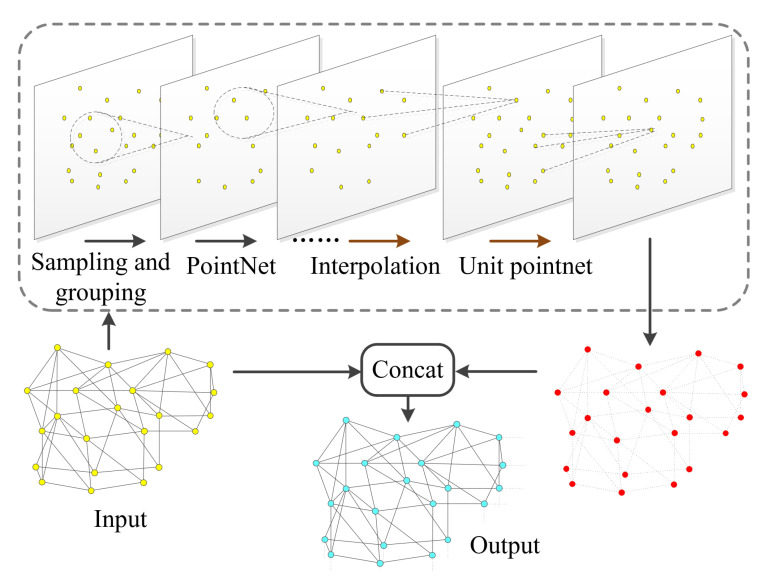
Schematic of the vertex feature learning.

To overcome the limitations above, we introduce an encoder–decoder neural network to learn the features in point cloud. The flowchart is depicted in Figure 2. The idea is to learn the features of the skeleton of the left myocardium at different receptive fields. Since the network uses a set of key points in the point cloud to represent the shape of the left myocardium, it exhibits good robustness to the noise in the point cloud, the absence of some points in the point cloud, etc. In the encoder layer, the initial vertices of mesh are regarded as a point cloud, and the key points and their features are extracted step by step through hierarchical downsampling, which is used to represent the significant features of the left myocardium. In the decoder layer, the key points restore the point cloud to the number of original vertices through interpolation processing and spread the key point features back to the original point set based on jump connections for generating the depth feature of the vertices, which contains the multilevel receptive field information of the triangular mesh to guide the mesh deformation effectively in the subsequent process.

Our vertex feature learning is similar to the encoding method of PointNet++ [21], which is an improved version of the PointNet network [16]. It can directly process the 3D point cloud and fuse the local and global features for object classification, component segmentation, scene segmentation and other tasks. Specifically, the learning structure consists of several point set abstraction levels. At each level, a set of points is extracted and abstracted to produce a new set with fewer elements by utilizing three key layers: a sampling layer, a grouping layer and a PointNet layer.

The sampling layer selects a set of points from input points, which defines the centroids of local regions. Considering the geometric character of the left myocardium, we use geometric subsampling instead of uniform sampling (such as iterative farthest point sampling) to choose a subset of points. The key idea of geometric subsampling is to pay more attention on the region with larger curvature. It consists of three steps. The first step is the pseudo-curvature estimation. The kNN neighborhood is built for each point and the angle between the center point and its neighbor is calculated. The larger the curvature in a region, the bigger the angles, so the angles can be taken as a coarse representative of curvatures in this region. The second step is region separation. Each point in the whole region is divided into two parts according to their angles. If the angles are larger than a threshold (such as 5 degrees), the corresponding points are put into a geometric region; otherwise, the points are put into a flat region. The third step is the respective uniform sampling in these regions. In comparison to iterative farthest point sampling, our proposed geometric subsampling can offer more points in the regions with significant geometric structure, while uniform sampling can be performed in local regions according to the requisite of the sampling number. Additionally, this sampling shows high stability and computational efficiency in comparison to pure curvature estimation and sampling.

The grouping layer intends to construct local region sets by adding local supportive features around each point in the subsampling set. For example, suppose *K* is the number of points in the neighborhood of a centroid point; after grouping, the size enlarges by *K* times, while each group corresponds to a local region.

The PointNet layer adopts a mini-PointNet to encode local region patterns into feature vectors. In this layer, each local region in the output is abstracted by its centroid and local feature that encodes the centroid’s neighborhood. In practice, the point-to-point relationship in the local region is captured by using relative coordinates together with point features. This kind of relationship can be used to guide the mesh deformation of the left myocardium.

After this hierarchical point set abstraction processing, the network extracts deep features on the sampling points with better coveragence of the entire point set given the same number of centroids (shown as red points in Figure 2). By concatenating the original point information, the vertex feature learning module outputs representative features associated with each point (shown as cyan points in Figure 2).

### 2.4. Iterated Mesh Deformation Module

#### 2.4.1. Residual Graph Convolution Network

Triangular mesh data can be regarded as a kind of graph-structured data set because the complex data dependencies exist among multiple entities or activities. It can therefore be organized and operated in a manner related to processing graph-structured data [10,22,23]. Recently, graph convolutional networks (GCNs) have been proposed to work on graph-structured data within the deep neural network paradigm [24,25]. The residual graph convolution operation [26,27] is also introduced to process the triangular mesh in our model. The basic formula of the graph convolution operation is

(1) $$f_{k}^{new}=ReLU\left(w_{0}f_{k}+\sum_{j\in N(k)}w_{1}f_{j}\right),$$

where $f_{k}$ is the feature of the *k*th vertex in the triangular mesh, N(k) is the neighborhood set of the *k*th vertex, $f_{k}^{new}$ is the learned feature of the *k*-th vertex after the graph convolution operation, and $w_{0}$ and $w_{1}$ are the parameters to be learned.

**Figure 3:**
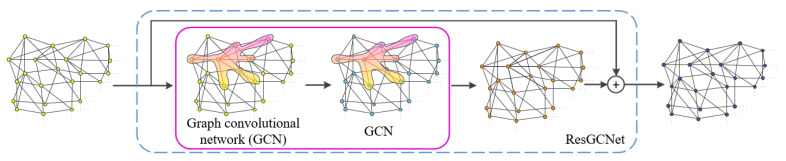
Flowchart of the residual graph convolutional network (ResGCNet).

The structure of the residual graph convolution network in our model is shown in Figure 3. The key idea relies on borrowing feature support in the neighborhood. Specifically, each vertex in the triangular mesh propagates its own features along the edges to the points in its neighborhood and borrows the features of the vertices in its neighborhood to refresh its own features, like the convolution operation in image data processing [28]. The neighborhood of each vertex in the triangular mesh is equivalent to the receptive field of each pixel in the feature map obtained by the image convolution operation. In the processing of image data, to obtain a more abstract feature map and obtain better results, the receptive field of each pixel in the map enlarges when increasing the depth of the neural network, so that the underlying features are more abundant. The convolution of the graph also adopts a similar strategy.

Increase in the depth of the graph convolutional neural network causes the features of each vertex in the triangular mesh to become more abundant. It can obtain better reconstruction results because the mapped underlying triangular mesh has more structural features. However, in image data processing, deepening the network structure is prone to gradient explosion. This problem can be solved by introducing skip layer connections into the network structure to form a residual structure. Motivated by this, a residual graph convolution by introducing skip layer connections based on the graph convolution structure is utilized in the proposed module. Specifically, as shown in Figure 3, the feature learning of the input triangular mesh is carried out by using the two-level convolution of graphs to update its vertex features, and then the updated triangular mesh features are connected with the input mesh features by residual convolution so as to obtain more abundant triangular mesh structural features.

#### 2.4.2. Iterated Deformation Module

**Figure 4:**
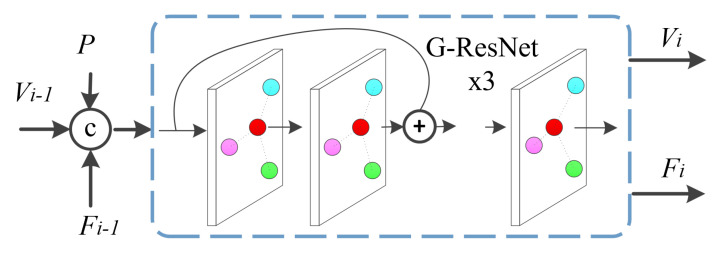
Composition of the *i*th mesh deformation module.

The residual graph convolution network designed in this paper includes three modules, each of which is composed of three residual graph convolutions and one graph convolution in series. The deformation result of the previous module is used as the input of the next module. To constrain the deformation of the triangular mesh so that the shape structure of the deformation result has little deviation from the left myocardium, the encoded features extracted by the vertex feature learning module from the initial triangular mesh is fed to each iterated deformation module. After that, the skeleton point cloud is up-sampled to obtain a feature point cloud with the same number of points as the initial triangular mesh vertices. In the experiments, the feature of each point in the point cloud is 128 dimensions. The feature point cloud is then fused with the vertices in the input triangular mesh of each module to obtain better deformation results. The composition of the deformation module is shown in Figure 4, where *P* represents the feature of each point in the mesh vertex set obtained by the vertex feature learning module; $V_{i-1}$, $F_{i-1}$ are the position and feature of each vertex of the output triangular mesh in the (i−1)th deformation module, respectively, and $V_{i}$, $F_{i}$ are their corresponding refined position and feature in the *i*th deformation module.

Specifically, the fusion features of the vertices of the input triangular mesh are sent to the graph convolution layer in advance for feature learning, and then the three cascaded residual graph convolution layers are fed for depth feature extraction so as to update the positions and features of the vertices of the triangular mesh. If only one deformation is carried out, the reconstruction results are not satisfactory. Therefore, the idea of iteration to take the current output triangular mesh as the next input is employed, and the result of vertex feature extraction is fused in the module. Then, the next generation of deformation with the new triangular mesh fusion feature as the input is performed. If this step is repeated, the resulting triangular mesh becomes angled towards the actual result for three-dimensional reconstruction of the left myocardium.

#### 2.4.3. Loss Function

It is difficult to directly compare the differences between two meshes. Therefore, similar to the mesh generation method exploiting geometric structure for graph-encoded objects [29], the same number of points in the reconstructed triangular mesh and mesh label surface is taken, and their differences are calculated to represent the difference between the meshes. Three loss functions are designed in this paper, namely the Chamfer distance, the normal vector distance, and the shape regularization [10]. Among them, the Chamfer distance is used to improve the similarity between point clouds, the normal vector distance is to make the surface of the object smoother, and the shape regularization is to prevent mesh degradation.

Suppose *P* and *G* are the point clouds sampled on the reconstructed grid and on the corresponding grid label, $n_{p}$ and $n_{g}$ are the normal vectors of point p∈P and g∈G, respectively, then the Chamfer distance and the normal vector distance are defined as follows:

(2) $$L_{c}\left(P,G\right)=\sum_{\left(p,g\right)\in N_{P,G}}{∥p-g∥}^{2}/\Omega \left(P\right)+\sum_{\left(g,p\right)\in N_{G,P}}{∥g-p∥}^{2}/\Omega \left(G\right),$$

(3) $$L_{n}\left(P,G\right)=-\sum_{\left(p,g\right)\in N_{P,G}}|n_{p}\cdot n_{g}|/\Omega \left(P\right)-\sum_{\left(g,p\right)\in N_{G,P}}\left|n_{g}\cdot n_{p}\right|/\Omega \left(G\right),$$

where Ω(P) and Ω(G) represent the number of points in the point clouds *P* and *G*, respectively. ∥p−g∥ and ∥g−p∥ both denote the distance between two points. For ∀p∈P, the closest g∈G has to be found and then the set of corresponding points in *P* can be represented as $N_{P,G}$. $N_{G,P}$ is defined in the same way. Specifically, $N_{P,G}=\left\{(p,\arg\min_{g}∥p-g∥):p\in P\right\}$; $N_{G,P}=\left\{(g,\arg\min_{p}∥g-p∥):g\in G\right\}$, so the expression for shape regularization is

(4) $$L_{e}\left(V,E\right)=\sum_{(v,v^{\prime })\in E}{∥v-v^{\prime }∥}^{2}/\Omega \left(E\right),$$

where *E* represents the edge set in the reconstructed triangular mesh, and Ω(E) is the number of edges. The mesh deformation network has a total of several deformation modules, and each deformation module has a corresponding loss function. The loss function of the deformation modules is the weighted sum of the above three similarity loss terms in each iteration:

(5) $$L_{loss}\left(P,G\right)=L_{c}+\lambda_{1}L_{n}+\lambda_{2}L_{e}.$$

In our experiments, the two weighting parameters $\lambda_{1}$ and $\lambda_{2}$ were empirically set.

## 3. Experimental Results and Discussion

### 3.1. Data Set and Experimental Platform

The data set in this experiment consisted of cardiac MR volumes of 20 subjects obtained from real clinical institutions. The acquisition equipment of these images included Siemens (Munich, Germany) (Avanto 1.5T, Espree 1.5T, Symphony 1.5T) and Philips (Amsterdam, The Netherlands) (Achieva 1.5T, 3.0T, Intera 1.5T). The image size ranged from 160× 288 to 512 × 512, and the number of slices along the z-axis of each subject ranged from 256 to 512. For each subject, the left ventricular myocardium tissue was labeled from images by two trained students. Their results were cross-validated by a clinical expert. The total labeling process requires nearly 50 h. In our experiments, 10 cases were randomly selected as the training data set and augmented to 80 cases by geometric operations such transformation, scaling and rotation. The augmented data set was then divided as follows: 60 were used for training, and the remaining 20 were used for validation. After these 10 subjects were randomly selected from the original data set, the remaining 10 subjects were used as the test data set.

The experimental platform was an Ubuntu 64-bit PC with a 2.0 GHz Intel CPU and a 48 GB RAM. The GPU was NVIDIA RTX 2080Ti with 11 GB of memory. The programming environment included Anaconda 5.0.1 (Python 3.6), TensorFlow1.4 and PyTorch1.2.0.

### 3.2. Evaluation Metrics

Because it is difficult to directly compare the difference between meshes, 90,000 points are sampled on the reconstructed triangular mesh and mesh labels. The difference between the two obtained point clouds is then used to represent the mesh difference. The point cloud sampled on the reconstructed triangular grid is called the predicted point cloud, while those sampled on the corresponding grid label are called the label point cloud. The evaluation metrics used in the experiments include the Chamfer distance (Chamfer), the normal consistency (Normal) and the ${F1}^{\tau }$ score [30].

To calculate the score, the precision $V_{precision}^{\tau }$ and recall $V_{recall}^{\tau }$ between the predicted point cloud and the labeled point cloud are first calculated. The accuracy rate $V_{precision}^{\tau }$ refers to the percentage of points whose distance from the label point cloud is less than the threshold τ in the predicted point cloud. The recall rate $V_{recall}^{\tau }$ refers to the percentage of points whose distance from the predicted point cloud is less than the threshold τ in the label point cloud. The ${F1}^{\tau }$ score is the harmonic mean of precision between $V_{precision}^{\tau }$ and recall $V_{recall}^{\tau }$, as shown in the following equation:

(6) $${F1}^{\tau }=\frac{2.0\times V_{precision}^{\tau }\times V_{recall}^{\tau }}{V_{precision}^{\tau }+V_{recall}^{\tau }+\delta },$$

where δ is a small constant to stabilize the calculation (e.g., $\delta =10^{-5}$). Among the above evaluation indicators, for the Chamfer distance, the smaller the value, the better the results. For the other two indicators, meanwhile, larger values indicate better results. However, these evaluation indicators cannot fully reflect the quality of the 3D reconstruction results. Therefore, to evaluate the quality of the reconstructed mesh more comprehensively, the experimental results were further analyzed with a qualitative score.

### 3.3. Parameter Settings

The implementation of the proposed model basically followed the backbone of the PointNet++ [21] and PointNet [16] networks. Although their tasks intrinsically differ from those of our model (classification and segmentation vs. reconstruction), their models present the graph convolutional neural network in a clear and efficient manner. The pre-trained model of PointNet++ was partially applied and the parameters were set to be as close as possible. Some key differences are stated as follows. In our experiments, the number of epochs was set to 15 and the batch size was one during the training process; that is, only one 3D segmentation result of the left myocardium was triangulated each time and sent to the mesh deformation network. In the network’s backward propagation computation, the Adam optimizer was used to update the parameters, in which the initial value of the learning rate was set to $5.0\times 10^{-5}$, and the updating method of the learning rate adopted a multi-step adjustment strategy.

**Figure 5:**
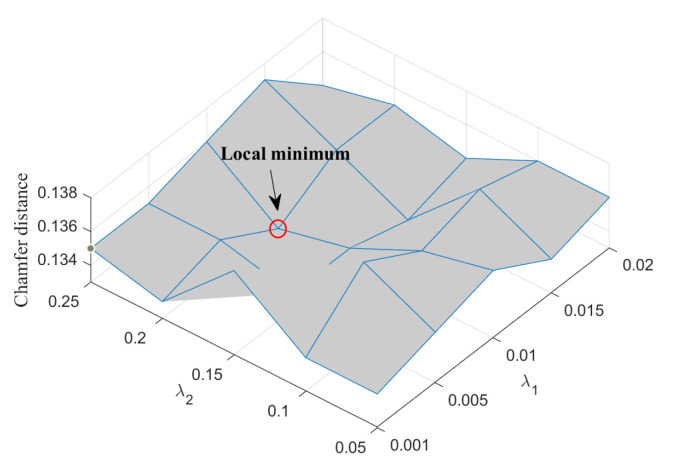
Weight settings of normal loss term $\lambda_{1}$ and shape regularization term $\lambda_{2}$.

Besides the parameters needed to be learned during the training, there are two key parameters (the weight $\lambda_{1}$ of normal loss term $L_{n}\left(P,G\right)$ and the weight $\lambda_{2}$ of shape regularization loss term $L_{e}\left(V,E\right)$) in the total loss function. Since the Chamfer distance loss function $L_{c}\left(P,G\right)$ is taken as the reference, we computed their values in the validation data set to estimate the coarse ratio and then chose $\lambda_{1}$ from the searching range of {0.001,0.005,0.01,0.015,0.02} and $\lambda_{2}$ from the searching range of {0.05,0.1,0.15,0.2,0.25}. Figure 5 presents the Chamfer distances corresponding to different parameters and it is shown that when $\lambda_{1}$ is 0.01 and $\lambda_{2}$ is 0.2, the Chamfer distance reaches its minimum, so they were chosen as the parameters in the following experiments.

### 3.4. Experimental Results and Analysis

#### 3.4.1. Overall Experiments and Analysis

To verify the effectiveness and characteristic of the proposed method, it was compared with three classical reconstruction algorithms, namely the marching cubes (MC) reconstruction algorithm [6,31], the Poisson surface reconstruction algorithm [32] and the Hermite radial basis functions (HRBFs) surface reconstruction algorithm [33,34]. MC is a well-known meshing algorithm to extract a polygonal mesh out of an isosurface from a three- or two-dimensional scalar field. It is primarily used for 3D modeling because of its simplicity. Considering that the original MC algorithm often produces many fragments of isosurfaces, we adopted an improved version with an adjacent lookup table and random sampling [35]. This serves as a baseline in our experiments.

The Poisson surface reconstruction algorithm is a famous implicit-function-induced reconstruction approach that solves for an approximate indicator formulation of the inferred solid whose gradient best matches the input normals. The output scalar function, represented in an adaptive octree, is then iso-contoured using adaptive marching cubes. It is a representative of fitting-based 3D reconstruction methods in our experiments.

The HRBF surface reconstruction algorithm is another well-known implicit-function-based method for reconstructing surfaces from scattered Hermite data points. A recent improved version is a closed-form formulation to construct HRBF-based implicitly by a quasi-solution to approximate the exact solution [34]. It is taken from an overall comparison for all these methods. This approach can automatically adjust the support sizes of basis functions to hold the error bound of a quasi-solution and then to generate an implicit function from positions and normals of scattered points without taking any global operation.

Table 1 reports the results of quantitative comparison among different reconstruction algorithms, where “*” indicates there is difference in mean between the proposed algorithm and the compared one in a *t*-test at a 95% significance level. The reconstruction effect of the proposed method is better than that of MC and Poisson models in the five metrics. It closes to that of Poisson in terms of the normal consistency (Normal), and HRBF in terms of the Chamfer distance (Chamfer), ${F1}^{0.3}$ and ${F1}^{0.5}$ evaluation index, which indicates that the proposed method is superior. From the number of vertices and the number of triangles in the 3D reconstruction results, it can be seen that the number of vertices and triangles in the reconstruction results of the Poisson algorithm and the HRBF algorithm is about two times that of the reconstruction results of the proposed method. This requires larger storage space and higher hardware performance.

**Figure 6:**
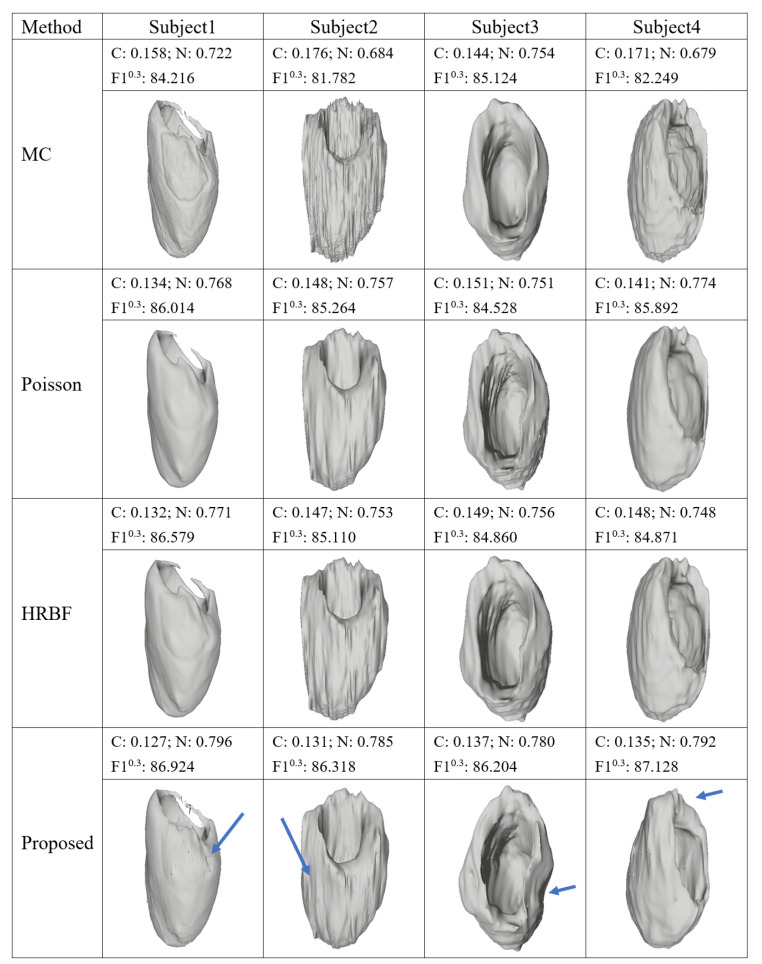
Examples of 3D left myocardium reconstruction results on four different individuals by compared algorithms, where the blue arrows denote the better reconstructed regions by our method. C: Chamfer distance; N: Normal value.

Figure 6 illustrates left myocardium reconstruction results of four individuals using the four compared algorithms. It is seen that they can all well express the geometric structure of the left myocardium as a whole. It can be seen that MC builds a much coarser result than other methods, especially on the second and fourth individuals: the surfaces have many parts full of non-smoothness that never appear in real cardiac tissues. In contrast, the Poisson and the HRBF appear more reasonable in these cases and their surfaces are remarkably smooth without sharp corners. However, in terms of the preservation of local geometric structure, our proposed method presents advantages. For instance, in the reconstruction of the third individual, the right part of the left myocardium by our method smoothly expresses the geometric variety while the other three methods produce some convex closure.

#### 3.4.2. Ablation Experiments and Analysis

The proposed method includes multiple functional modules such as deformation, point deep feature extraction and residual graph convolution networks. To verify the rationality and functionality of each module, three variant methods were built by removing the deformation, deep feature learning and residual connection in ResGCNet modules independently. Three ablation experiments were then carried out under identical experimental conditions.

Table 2 reports the quantitative comparative reconstruction results of these three variant methods and the proposed one. The proposed method has the smallest Chamfer distance, followed by Variant3 that removes the residual graph convolution network module, and the worst is Variant2 that removes the deformation module. Regarding the Normal score, the proposed method obtains the largest measure, while Variant2 obtains the smallest, indicating that the deformation module can guide the deformation of the triangular mesh.

**Figure 7:**
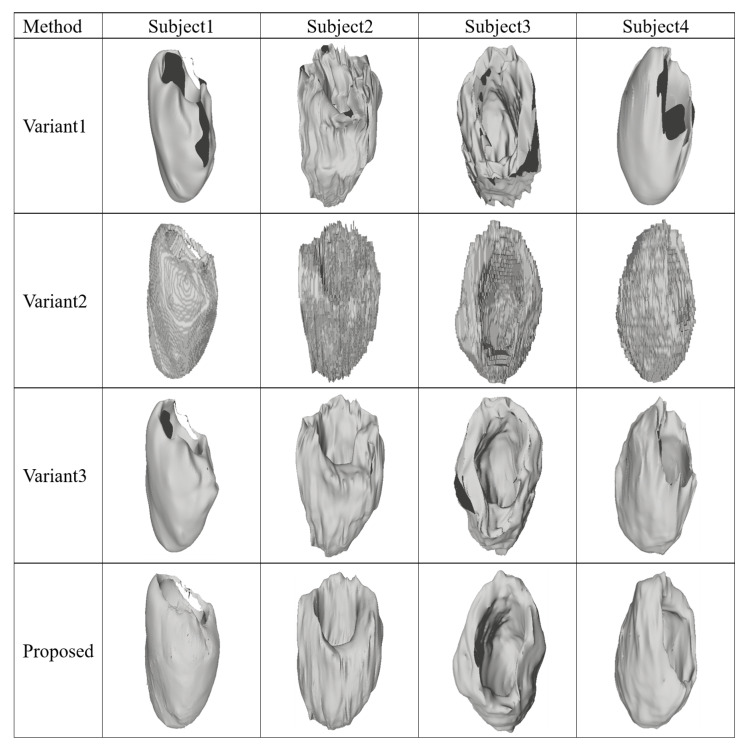
Examples of 3D reconstruction results of the proposed method and three variants.

Figure 7 further illustrates the 3D reconstruction results of the three variants and the proposed one. It can be seen that after removing the point deep feature extraction module, the reconstruction result cannot maintain the complete shape and structure of the left myocardium well. This module processes the point cloud of the initial triangular mesh to obtain the skeleton feature of the point cloud, which has better robustness to the lack of points in the point cloud and external noise. Since the point cloud of the initial triangular mesh can roughly express the three-dimensional structural features of the left myocardium, the skeleton feature of point cloud can be used to constrain the deformation of triangular mesh, so that the deformation does not cause a large deviation. To obtain a better reconstruction result, the obtained result after removing the deformation module is the initial triangular mesh. Since the initial triangular mesh is directly obtained from the 3D segmentation result of the cardiac images, it has the shape and structure characteristics of the left myocardium. However, the initial triangular mesh does not reflect the surface information of the left myocardium with great fitness, which is also a disadvantage of using voxels to represent the 3D reconstruction results.

The reconstruction results by removing residual connections are similar to those obtained from the full model, but there are still some differences. When deepening the network structure, the use of residual connections can prevent exploding gradients. In image data processing with regular structure, deepening the network structure can increase the receptive field of a pixel and make the underlying features mapped by the pixel more abundant so as to obtain better results. Here, by deepening the network structure, the features represented by each vertex in the triangular mesh are more abundant, and more structural features of the underlying triangular mesh are mapped to obtain better reconstruction results. Meanwhile, a graph residual convolution module is added to the network structure to avoid gradient explosion.

#### 3.4.3. Qualitative Experiments on Surface Refinement

The proposed method can be coarsely regarded as an improvement of initial iso-surface mesh by designing an iterated residual graph convolutional neural network to drive the mesh deformation. To investigate the rationale of the proposed mesh deformation, a qualitative experiment was further conducted to evaluate the difference between the initial triangular mesh and the final mesh. Considering that the ground truth of reconstruction is difficult to obtain, their distances were directly computed as a form of histogram and displayed as different colors on the final mesh.

**Figure 8:**
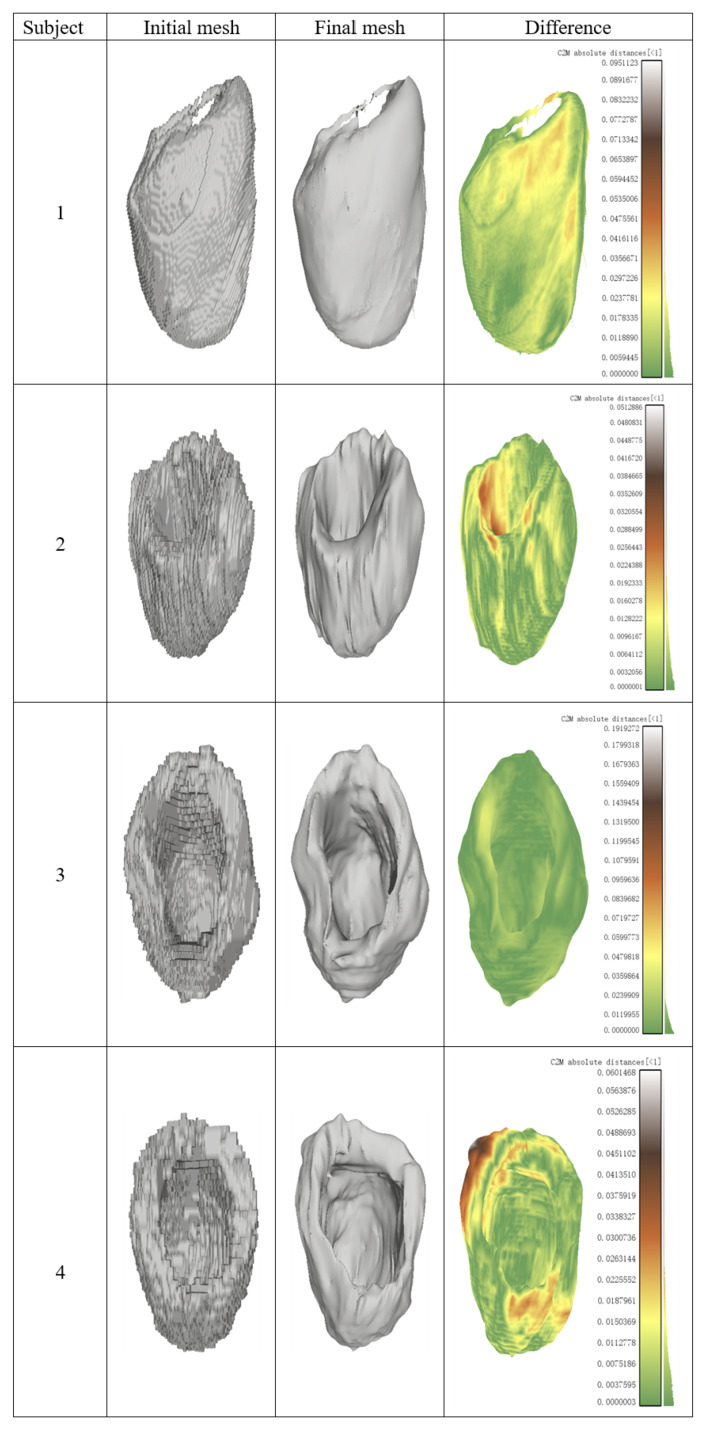
Examples of reconstruction difference between the initial mesh, the final mesh by proposed method and their distribution of distances.

Figure 8 demonstrates the experimental results on four individuals. The initial reconstruction surfaces in the left column are coarse and full of voxel-wise steps, while after the mesh deformation processing by iterated residual graph convolution network, the final surfaces look much more smooth and the geometric structures are well preserved. Their difference in the right column is consistent in most regions, indicating that the proposed method improves the mesh quality in a global way and has remarkable improvement on the initial 3D reconstructed mesh.

**Figure 9:**
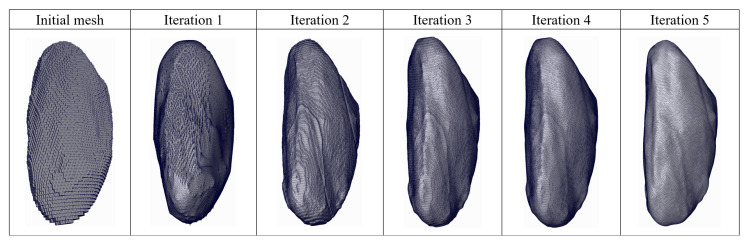
Example of iterated deformation process from the initial mesh to the final shape by the proposed method.

Figure 9 demonstrates an example of iterated deformation process from the initial mesh by the proposed algorithm in another viewpoint. The shape of left myocardium is hardly observed from the initial reconstruction mesh in the left column by the Cubify algorithm. The details are totally missing and only a rough outline can be inferred. After the first iteration by the mesh deformation processing using iterated residual graph convolution network, some regions have changed to present a similarity to the realistic surface; however, there are many sharp and matte regions. During the second, third and fourth iterations, the number of these regions is gradually decreasing, while the whole region becomes increasingly smoother and the positions of vertices have more uniformity in comparison to the results by the previous iterations. From this example, it can be seen that the vertices are adjusted over iterations, and deep residual graph convolution network jointly with vertex feature learning can greatly contribute to the final reconstructed shape.

#### 3.4.4. Computational Cost

The proposed method consists of several parts such as segmentation, Cubify, iterated deformation and different parts that require different time to implement. During the experiment, the computational cost of each part is performed under the same experimental conditions as much as possible. The results of average test time by compared methods are reported in Table 3:

As can be seen from the table above, the reconstruction speed of the proposed algorithm achieved a leading advantage with 2614 ms and the iterated deformation taking about 56.5% of the time. In particular, the Cubify initial reconstruction takes less than one second than the improved MC algorithm that takes about 6200 ms. In traditional methods, HRBF achieves more accurate performance than MC and Poisson, but with a computational cost of nearly 59,000 ms, significantly longer than MC’s 6200 ms and Poisson’s 2800 ms. The main reason for this expensive time cost is due to the HRBF interpolant. The performance can be improved by using the compactly-supported RBFs that change the global interpolation effect of the interpolant [33]. In contrast, the proposed algorithm has rather high computational efficiency due to the intrinsic characteristic of deep residual graph convolution learning technique. The three variant algorithms also require less time than traditional methods, and Variant1 only requires almost 1400 ms to complete 3D reconstruction, remarkably less than the other two variants. However, its reconstruction quality is much worse because it does not incorporate the iterated deformation module.

#### 3.4.5. Limitations and Improvement Directions

In the vertex feature learning module, a feature extraction structure similar to PointNet++ is employed. Due to the difference in the structure of the left myocardium, there may be uneven density distribution for the point cloud formed by the vertices of the input triangle mesh, which causes the important features of some key points to be lost during the processes of sampling and grouping. In the future, adaptive density learning strategy could be introduced to assign more suitable scale neighborhoods to key points in the grouping process so as to achieve better feature extraction results. This will be beneficial in guiding the deformation of the triangular mesh in subsequent steps to achieve better 3D reconstruction results of the left myocardium.

## 4. Conclusions and Future Work

This paper presents a deep learning method for personalized 3D reconstruction of the left myocardium from MRI images. To better handle the variety across individuals, the algorithm directly obtains the corresponding initial triangular mesh from the 3D segmentation results of left myocardium images. Then, a residual graph convolutional neural network is designed to refine the initial triangular mesh in an iterated manner. The learned vertex features that encode the local region are incorporated to the mesh deformation network at each iteration so as to assist the deformation towards the right direction. As a result, the reconstructed surface is a geometrically valid model meeting the requirements of personalized left myocardium. Experimental results validate the performance of the proposed approach with quantitative and qualitative comparison to some state-of-the-art algorithms.

It should be noted that the quality of the left myocardial segmentation results can directly affect the performance of the final reconstruction. Although the powerful deep-learning-based segmentation approach is employed, the segmentation and reconstruction are separated processing steps in the proposed model. In future study, the 3D segmentation and 3D reconstruction of the left myocardium will be integrated as a whole to improve the reconstruction results.

## Figures and Tables

**Table 1:** Quantitative comparison of the reconstruction results of different algorithms.

Method	Chamfer	Normal	F1${}^{0.1}$	F1${}^{0.3}$	F1${}^{0.5}$	Ω(V)	Ω(F)
MC	0.164 *	0.718 *	34.654 *	83.717 *	92.665 *	43,431	86,822
	±0.034	±0.05	±1.478	±1.319	±1.344	±1348	±2712
Poisson	0.137 *	0.770 *	36.904 *	85.167 *	94.249 *	178,821	357,628
	±0.001	±0.007	±0.691	±0.589	±0.842	±17,985	±35,984
HRBF	0.136	0.766 *	37.486 *	85.852	95.489	271,478	107,455
	±0.001	±0.007	±0.95	±0.768	±0.791	±40,197	±49,747
Proposed	0.133	0.789	39.362	86.058	95.834	45,769	91,078
	±0.004	±0.005	±0.848	±0.915	±0.682	±1416	±2547

${F1}^{\tau }$: The harmonic mean of precision between $V_{precision}$ and recall $V_{recall}$ with a threshold τ. Ω(·): The number of points in a point cloud.

**Table 2:** Quantitative comparison of the reconstruction results of three variants and the proposed method.

Method	Module			Chamfer	Normal	F1${}^{0.1}$	F1${}^{0.3}$	F1${}^{0.5}$
	**Deformation**	**Deep Feature**	**ResNet**					
Variant1	✗	✓	✓	0.804	0.606	7.285	31.37	54.06
				±0.056	±0.007	±0.235	±1.174	±1.206
Variant2	✓	✗	✓	2.011	0.578	6.269	25.737	46.191
				±0.173	±0.03	±0.34	±1.188	±1.359
Variant3	✓	✓	✗	0.294	0.637	30.499	80.285	89.135
				± 0.07	±0.027	±1.339	±1.681	±1.175
Proposed	✓	✓	✓	0.133	0.789	39.362	86.058	95.834
				±0.004	±0.005	±0.848	±0.915	±0.682

**Table 3:** Average reconstruction time by the proposed method and compared algorithms (ms/subject).

	Part of Proposed Method			MC	Poisson	HRBF	Variant1	Variant2	Variant3
	**Segmentation**	**Cubify**	**Deformation**						
Test time	526	610	1478	6218	28,476	59,374	1427	2148	2295

## Data Availability

The processed data are available upon request to the corresponding author.
